# Anti-PD-1 Therapy with Adjuvant Ablative Fractional Laser Improves Anti-Tumor Response in Basal Cell Carcinomas

**DOI:** 10.3390/cancers13246326

**Published:** 2021-12-16

**Authors:** Uffe Høgh Olesen, Martin Wiinberg, Catharina Margrethe Lerche, Ditte Elisabeth Jæhger, Thomas Lars Andresen, Merete Haedersdal

**Affiliations:** 1 Department of Dermatology, Copenhagen University Hospital—Bispebjerg and Frederiksberg, Nielsine Nielsens Vej 17, 2400 Copenhagen, Denmark; Catharina.Margrethe.Lerche@regionh.dk (C.M.L.); mhaedersdal@dadlnet.dk (M.H.); 2 Department of Health Technology, Technical University of Denmark, 2800 Kongens Lyngby, Denmark; marwii@dtu.dk (M.W.); dija@dtu.dk (D.E.J.); tlan@dtu.dk (T.L.A.); 3 Department of Pharmacy, University of Copenhagen, 2100 Copenhagen, Denmark

**Keywords:** ablative fractional laser, programmed cell death-1 inhibitor, basal cell carcinoma, autochthonous cancer model, immunotherapy

## Abstract

**Simple Summary:**

In some mouse models, ablative fractional laser (AFL) enhances the efficacy of anti-programmed cell death1 therapy (aPD-1), which was recently approved for basal cell carcinoma (BCC). In this explorative study, we aimed to assess locally applied AFL as an adjuvant to systemic aPD-1 treatment in a clinically relevant BCC model. BCC-carrying mice received aPD-1 alone, AFL alone, aPD-1+AFL, or no treatment. Both aPD-1 and AFL alone significantly increased survival time relative to the untreated controls, while aPD-1 that had been complemented with AFL further promoted survival and improved tumor clearance and growth rates. The BCCs were poorly immune infiltrated, but aPD-1 with adjuvant AFL and AFL alone induced substantial immune cell infiltration in tumors and increased the levels of relevant immune cell subtypes. Thus, the anti-tumor response that was generated by aPD-1 with adjuvant AFL may potentially be promoted by increased immune activity in tumors. In conclusion, the use of a local AFL adjuvant to systemic aPD-1 therapy could hold substantial promise for BCC treatment.

**Abstract:**

The efficacy of anti-programmedcelldeath1therapy (aPD-1), which was recently approved for basal cell carcinoma (BCC) treatment, can be enhanced by adjuvant ablative fractional laser (AFL) in syngeneic murine tumor models. In this explorative study, we aimed to assess locally applied AFL as an adjuvant to systemic aPD-1 treatment in a clinically relevant autochthonous BCC model. BCC tumors (*n* = 72) were induced in Ptch1^+/−^K14-CreER2p53^fl/fl^-mice (*n* = 34), and the mice subsequently received aPD-1 alone, AFL alone, aPD-1+AFL, or no treatment. The outcome measures included mouse survival time, tumor clearance, tumor growth rates, and tumor immune infiltration. Both aPD-1 and AFL alone significantly increased survival time relative to untreated controls (31 d and 34.5 d, respectively vs. 14 d, *p* = 0.0348–0.0392). Complementing aPD-1 with AFL further promoted survival (60 d, *p* = 0.0198 vs. aPD-1) and improved tumor clearance and growth rates. The BCCs were poorly immune infiltrated, but aPD-1 with adjuvant AFL and AFL alone induced substantial immune cell infiltration in the tumors. Similar to AFL alone, combined aPD-1 and AFL increased neutrophil counts (4-fold, *p* = 0.0242), the proportion of MHCII-positive neutrophils (*p* = 0.0121), and concordantly, CD4^+^ and CD8^+^ T-cell infiltration (*p* = 0.0061–0.0242). These descriptive results suggest that the anti-tumor response that is generated by aPD-1 with adjuvant AFL is potentially promoted by increased neutrophil and T-cell engraftment in tumors. In conclusion, local AFL shows substantial promise as an adjuvant to systemic aPD-1 therapy in a clinically relevant preclinical BCC model.

## 1. Introduction

Basal cell carcinoma (BCC) is the major subtype of keratinocyte cancer; it is the most prevalent human cancer worldwide, with an estimated 5.4 million cases in the US alone [1]. Incidence rates have been steadily increasing for decades, and health care costs are substantial [2,3]. Surgery is the first-line treatment for localized tumors, while the current options for pharmacological therapies show limited efficacy [4,5].

Anti-programmed cell death-1 immune checkpoint therapy (aPD-1) is a cancer immunotherapy that has revolutionized cancer treatment regimens in recent years and is of interest for the treatment of complex keratinocyte carcinoma cases. Functionally, aPD-1 promotes an adaptive anti-tumor immune response by blocking the tumor-induced suppression of immunosurveillance [6]. The efficacy of aPD-1 has been shown to correlate to the mutational burden of tumors [7,8]. Thus, a high mutation rate increases the likelihood of generating immunogenic neoantigens and concordantly inducing tumor-specific T-cells that can be reinvigorated by aPD-1 therapy [7,8,9]. Keratinocyte cancers predominantly originate from sun-damaged skin and often present a high mutational burden, which makes them a promising target for aPD-1 therapy [10]. In accordance with this, the aPD-1 drug cemiplimab was FDA approved in 2018 for the treatment of locally advanced and metastatic squamous cell carcinomas, another subtype of keratinocyte cancer [11]. Importantly, cemiplimab was also recently approved for locally advanced and metastatic BCC. The potential of aPD-1 to treat BCC is supported in the literature [12,13,14,15,16] and is currently being further explored in at least two clinical trials (Clinicaltrials.gov Identifier NCT03132636 and NCT02834013). However, a substantial clinical response to aPD-1 has only been observed in a subset of treated patients; thus, it is important to identify adjuvant treatments that can act synergistically to increase treatment response rates [17].

Ablative fractional laser (AFL) is a well-established modality in dermatology that induces a grid of microscopic treatment zones of ablated tissue that are surrounded by the thermally coagulated tissue in the treated skin [18]. AFL exposure, partially due to the thermal injury to skin cells, stimulates a substantial anti-tumor immune response, including recruitment of neutrophils and antigen-specific cytotoxic T-cells [10,19,20]. This process involves the induction of immunogenic cell death followed by the release of damage-associated molecular patterns (DAMPs) and potential neoantigens, resulting in the activation of innate and adaptive immune cells [21,22,23,24,25]. In accordance with this, AFL has been shown to stimulate the local infiltration of immune cells in healthy human skin by increasing the levels of immune-attracting cytokines and growth factors, including IL-6, transforming growth factor-β, basic fibroblast growth factor, and platelet-derived growth factor [26,27]. Further, in murine syngeneic tumor models (i.e., subcutaneous-inoculated tumor models), AFL has been shown to induce tumor-specific cytotoxic T-cells and adaptive anti-tumor immunity [19,20]. Finally, AFL has been reported to boost the tumor response of immunotherapy, including aPD-1 [19,20,24] and the Toll-like receptor agonist imiquimod in preclinical studies [28]. 

We hypothesized that treating tumors locally with AFL could serve as an adjuvant to systemically administered aPD-1 and that it could substantially improve its therapeutic efficacy in BCCs. Hence, using a translational study, we aimed to confirm a potential increase in the efficacy and to form the rationale for further clinical investigations. This was achieved by exploring the impact of aPD-1 treatment with adjuvant AFL on anti-tumor response, including mouse survival time, tumor clearance and tumor growth rates, and immune infiltration in a clinically relevant murine BCC model.

## 2. Results

### 2.1. Tumor Response

**Figure 1:**
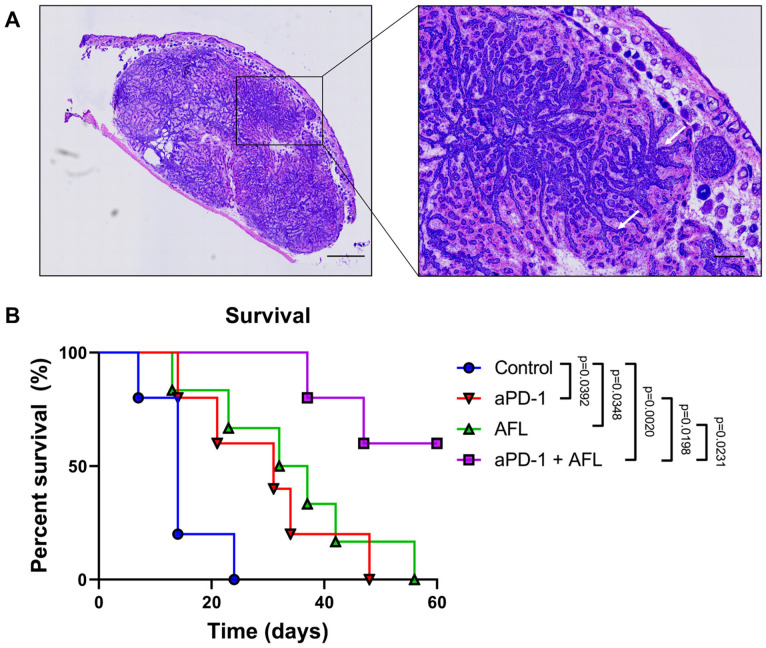
Histological presentation of murine BCCs and treatment-dependent increases in survival time (**A**) H&E-stained section of a nodular murine BCC showing characteristic basaloid lobules with peripheral nuclear palisading (arrows). (**B**) Kaplan–Meier survival plot displaying time from treatment initiation (time = 0) until a humane endpoint is reached (i.e., largest tumor size ≥865 mm^3^). Size bars: 1000 µm and 200 µm for overall and close-up images, respectively. aPD-1: programmed cell death-1 immune checkpoint inhibitor. AFL: ablative fractional laser. Group sizes: *n* = 5–6 mice.

All three treatment interventions, including aPD-1 monotherapy, AFL monotherapy, and aPD-1 with adjuvant AFL, showed improved tumor response, including mouse survival time, tumor clearance, and tumor growth. The median survival time was increased by both aPD-1 and AFL individually from 14 days for the control group to 31 and 34.5 days, respectively (Figure 1, *p* = 0.0348–0.0392). Adjuvating aPD-1 with AFL further increased survival compared to either monotherapy, reaching a median survival of 60 days (*p* = 0.0198–0.0231).

**Figure 2:**
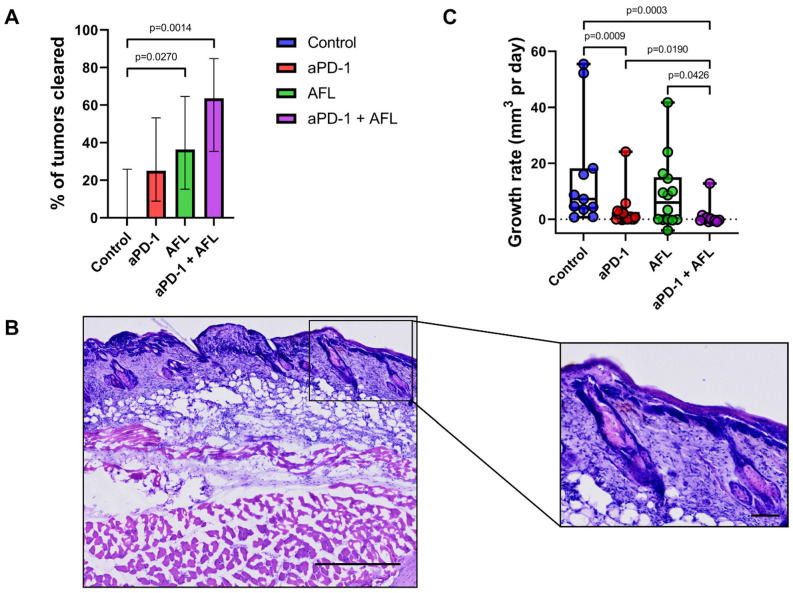
Improved tumor growth rates and tumor clearance achieved by aPD-1 adjuvated with AFL (**A**) Percentage of treated tumors cleared during the study period displayed with standard errors. (**B**) H&E-stained section of skin at the location of a cleared tumor. (**C**) Median tumor growth rates of treated BCC tumors with box (interquartile range) and max-min whiskers. aPD-1: programmed cell death-1 immune checkpoint inhibitor. AFL: ablative fractional laser. Group sizes *n* = 11–17 tumors. Size bars: 500 µm and 100 µm for overall and close-up images, respectively.

Regarding tumor clearance, aPD-1 with adjuvant AFL reached a tumor clearance rate of 64% (*p* = 0.0014 vs. untreated control, Figure 2A,B), although aPD-1 alone did not significantly increase clearance rates. On the other hand, AFL alone resulted in improved tumor clearance (36%, *p* = 0.0270) that was not significantly lower than that of aPD-1 and AFL combined (*p* = 0.2008).

Conversely, the growth rates of the aPD-1-treated tumors were significantly lower compared to untreated tumors (*p* = 0.0009, Figure 2C), whereas AFL monotherapy did not significantly reduce tumor growth (*p* = 0.2385). Yet, complementing aPD-1 with AFL further decreased tumor growth rates compared to either treatment alone (*p* = 0.0190–0.0426), and aPD-1 with AFL adjuvant was the only intervention to display a negative median tumor growth rate.

### 2.2. Tumor Immune Infiltration

**Figure 3:**
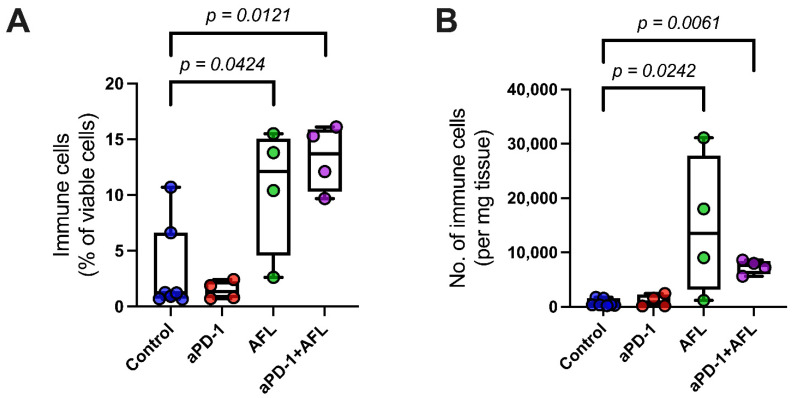
AFL as monotherapy and aPD-1 with adjuvant AFL induce a general increase in tumor immune cell infiltration (**A**) Proportion of immune cells (CD45^+^ viable cells) of the total number of viable single cells extracted from tumors. (**B**) Absolute number of immune cells per mg of tumor tissue. aPD-1: programmed cell death-1 immune checkpoint inhibitor. AFL: ablative fractional laser. Group size *n* = 4–7.

The BCC tumors demonstrated low overall immune filtration, with immune cells representing only 1.2% of the total viable cell count when examined by flow cytometry (Figure 3A). AFL produced a pronounced immune infiltration, both as a monotherapy and as an aPD-1 adjuvant, while aPD1 alone did not increase immune cell levels (Figure 3A,B). Compared to the untreated controls, the aPD-1 with adjuvant AFL significantly increased the frequency of the CD45^+^ immune cells relative to the total count of viable cells (*p* = 0.0121). This corresponds to a 10-fold increase in the number of CD45^+^ immune cells per milligram of tumor tissue compared to the control group (*p* = 0.0061).

**Figure 4:**
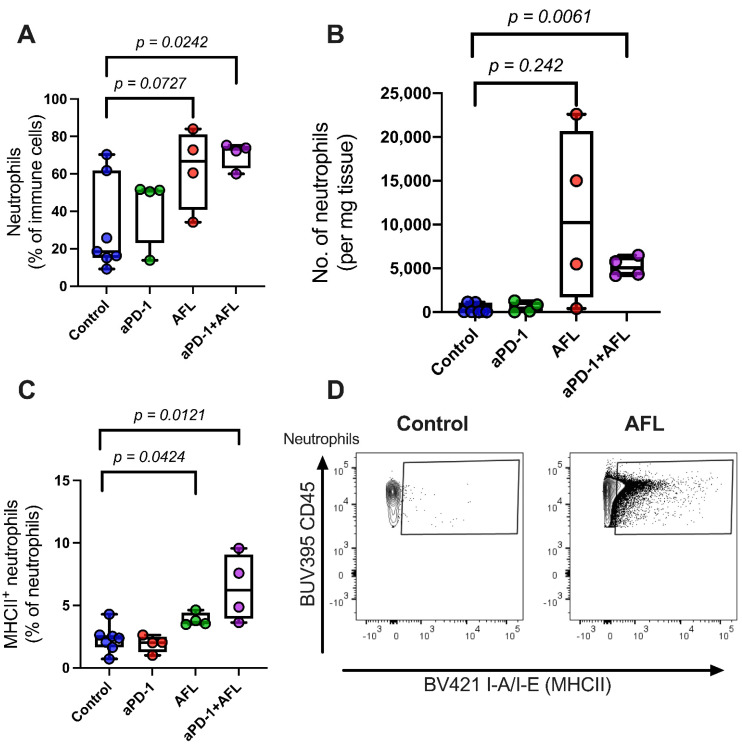
Neutrophil tumor infiltration and major histocompatibility complex class II expression on tumor-infiltrating neutrophils enhanced by aPD-1 with AFL and AFL alone. Neutrophil infiltration relative to (**A**) total immune cell counts (in percentage) or (**B**) tumor weight (number of cells per mg tumor). (**C**) Major histocompatibility complex class II^+^ (MHCII) neutrophils out of all neutrophils (determined by the polymorphic determinant I-A/I-E). (**D**) Representative contour plots of MHCII signal on gated neutrophils. Neutrophils are defined as CD45^+^CD11b^+^CD11c^−^Ly-6C^int^Ly-6G^+^ cells. aPD-1: programmed cell death-1 immune checkpoint inhibitor. AFL: ablative fractional laser. Group size *n* = 4–7.

Further analysis of the immune cell subpopulations revealed that aPD-1 treatment with adjuvant AFL resulted in a four-fold increase in the relative proportion of neutrophils (*p* = 0.0242, Figure 4A) and a significant increase in the total number of neutrophils per milligram tumor of tissue (*p* = 0.0061) compared to the untreated controls (Figure 4B). Accordingly, neutrophils constitute more than 60% of all CD45^+^ immune cells in tumors treated with aPD-1 and AFL combined. In addition, the proportion of neutrophils expressing major histocompatibility complex class II (MHCII) was significantly increased in both groups that received AFL treatment compared to the untreated controls (*p* = 0.0121–0.0424), Figure 4C,D).

**Figure 5:**
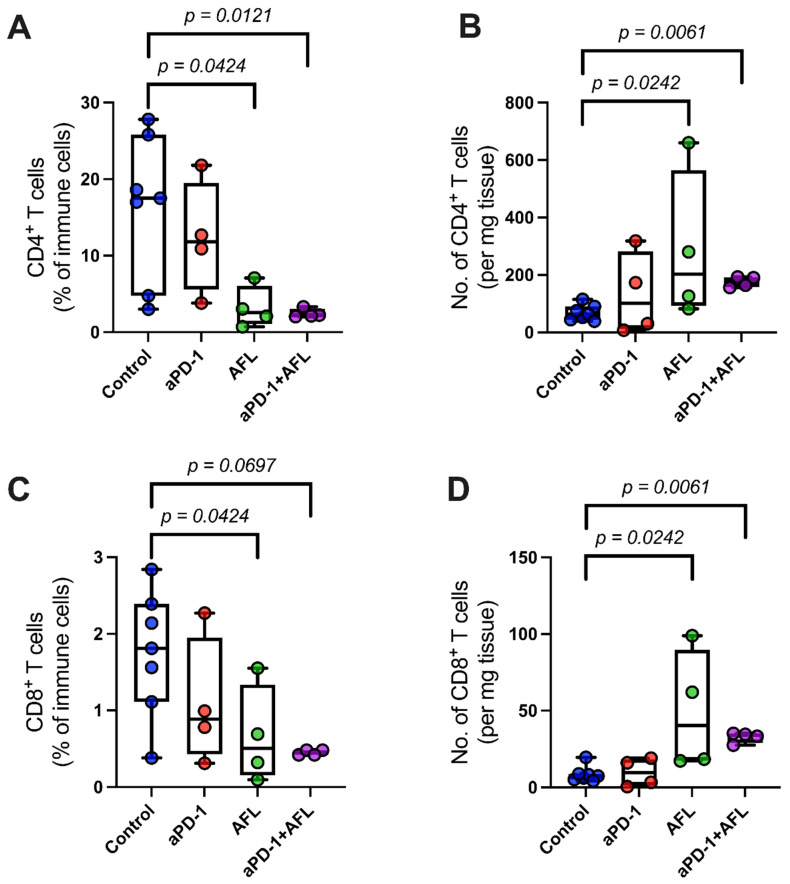
Absolute numbers of tumor-infiltrating lymphocytes are increased by aPD-1 with adjuvant AFL and AFL alone. Number of CD4^+^ and CD8^+^ T-cells relative to (**A**,**C**) total immune cell counts (in percentage) and (**B**,**D**) relative to tumor weight (number of cells per mg tumor). aPD-1: programmed cell death-1 immune checkpoint inhibitor. AFL: ablative fractional laser. Group size *n* = 4–7.

With regard to the adaptive immune system, the untreated BCCs generally displayed low levels of tumor-infiltrating lymphocytes relative to the total immune cell counts. The tumor-infiltrating lymphocytes primarily consisted of CD4^+^ T-cells with a very small percentage of CD8^+^ T-cells (Figure 5). Both aPD-1 with AFL and AFL alone induced an increase in the absolute CD8^+^ and CD4^+^ T-cell numbers in the tumors (*p* = 0.0061–0.0242) even though the proportions of the CD8^+^ and CD4^+^ T-cells relative to the total immune population were decreased—this was possibly due to the substantial neutrophil recruitment (Figure 5). In addition, the ratio of CD4^+^ to CD8^+^ T-cells showed a non-significant tendency to decrease in response to aPD-1 combined with AFL, suggesting a shift in the T-cell profile (Figure S1).

The T-cell counts were found to be too low to assess the immune-suppressive T-cell subpopulation and their response to treatment. Nonetheless, no increase in the percentage of monocytic myeloid-derived suppressor cells (Mo-MDSC) out of the total immune cell counts was observed for any treatment intervention, although the levels of Mo-MDSC relative to tumor weight were elevated for AFL alone and for aPD-1 with adjuvant AFL (*p* = 0.0061, Figure S2A,B). In addition, no significant changes in the frequency of the MHCII expressing Mo-MDSCs or in the median fluorescence intensity of the MHCII that were expressed were seen across interventions, indicating no changes in the phenotype of Mo-MDSC (Figure S2C,D).

## 3. Discussion

This study is the first to demonstrate a significant impact of adjuvant AFL on aPD-1 treatment for BCC tumors in a clinically relevant autochthonous murine model [29] and suggests a role of local AFL exposure to BCC tumors in boosting the tumor response to systemic aPD-1 treatment. While both aPD-1 and AFL increased survival time compared to untreated controls when they were used as monotherapies, complementing aPD-1 treatment with AFL significantly improved survival time and tumor growth rates compared to either treatment alone, and the combination treatment obtained the highest cleared tumor proportion of any of the treatment interventions. Thus, AFL may act synergistically to improve the BCC tumor response to aPD-1 treatment.

These results are consistent with the existing literature on AFL as an adjuvant for aPD-1 since the efficacy of the combination has previously been reported in a murine syngeneic subcutaneous-inoculated tumor model using CT26 colon cancer cells [19,20]. Using a similar treatment strategy, the authors showed how a single exposure to AFL substantially increased tumor clearance in response to systemic aPD-1. Additionally, they reported that AFL leads to a potent CD8^+^ T-cell and neutrophil anti-tumor immune response and the induction of systemic anti-tumor immunity that likely contributed to tumor clearance in this highly immunogenic model [30].

Similarly, we previously documented an adjuvant effect of AFL on topically applied immunotherapy, namely imiquimod, a Toll-like receptor 7-agonist immunotherapeutic drug that is commonly used for keratinocyte carcinoma treatment [28]. We reported a substantial increase in tumor clearance and lymphocyte infiltration when using AFL as an adjuvant for imiquimod in an autochthonous squamous cell carcinoma model, underlining the potential of AFL as an adjuvant to immunotherapeutic treatments.

To explore the possible role of the immune system in our findings on tumor response, we completed a descriptive characterization of the immunological response to the different treatments. Immune infiltration was remarkably low in untreated tumors (a median of 1.2%) compared to common syngeneic murine tumor models [31]. Crucially, substantial immune cell infiltration in response to AFL alone and as an adjuvant for aPD-1 was observed, and that was driven to a high degree by increased numbers of neutrophils. While the impact of tumor-infiltrating neutrophil levels is debated [32,33,34], the importance of the neutrophil phenotype and the ability of treatments to polarize neutrophils from a pro-tumor to an anti-tumor phenotype has been emphasized in previous studies [35,36,37]. Noticeably, AFL has been shown to repolarize tumor-associated neutrophils towards an anti-tumor phenotype [20], and a strong neutrophil response to AFL treatment has been documented in a number of preclinical models [19,20,28,38]. Moreover, it has been shown that AFL-induced neutrophil infiltration can be involved in activating cytotoxic CD8^+^ T-cells, playing an important role in anti-tumor immune response [19,20]. Interestingly, we found that the fraction of neutrophils presenting MHCII molecules on the surface was doubled upon combined aPD-1 and AFL treatment. This MHCII expression suggests that the neutrophils participate in a potential antigen-presenting activity, possibly priming naïve CD4^+^ T-cells [39,40,41,42].

Consistently, an increased number of tumor-infiltrating T-cells was observed following AFL treatment, indicating that the inflammation caused by AFL mediates the recruitment of more T-cells to the tumor. Further, while the engrafted lymphocytes mainly consisted of CD4^+^ T-cells in untreated tumors, treatment with aPD-1 and adjuvant AFL led to a relative increase in the CD8^+^ T-cell population, suggesting a shift in the T-cell profile, which is in accordance with previous reports [19,28].

The autochthonous Ptch1^+/−^ K14-CreER2 p53^fl/fl^ murine model is a clinically relevant BCC model that develops tumors with characteristics analogous to human BCC tumors [43]. While we have not studied the expression levels of PDL-1 or PDL-2 in this model, there is clinical evidence to support increased PDL-1 expression in a significant portion of BCC tumor cells and tumor-infiltrating immune cells [44,45,46]. Furthermore, the BCCs developed in Ptch1^+/−^ models are very heterogeneous [47], similar to BCC tumor presentation in a clinical setting. The tumor heterogenicity that is due to the autochthonous nature of the model also presents limitations. The time to tumor following induction varies substantially, and common tumor neoantigens have not been identified, complicating the mechanistic characterization of anti-tumor immune responses. The group sizes used in this study correspond to the sizes of the groups used in previous treatment studies using the same model (mice per group *n* = 4–5, tumors per group *n* = 6–10) [48,49].

Another potential study limitation may be that immunological analysis was performed on day five after treatment initiation even though the levels of cytotoxic CD8^+^ T-cells may peak at a later time point as the one previously found for AFL treatment in a model of squamous cell carcinomas [28]. Thus, it is possible that the tendency of a shift in the T-cell profile could have been even clearer following the full aPD-1 treatment regimen (5 injections vs. 3 injections). Similarly, our results do not show a significant increase in neutrophils, lymphocytes, or overall immune infiltration in the AFL+aPD-1 group compared to the group treated with AFL alone. The lack of significantly increased immune infiltration 5 days after aPD-1 treatment was initiated has previously been reported despite having a significant impact on tumor response and systemic immunity [20]. Another limitation is that in the study, an isotype antibody was not applied to the control groups. Finally, within the scope of the study, it was not possible to determine if the increased CD8^+^ T-cell population was tumor antigen-specific. However, the prolonged survival of the mice following aPD-1 adjuvated with AFL suggests that adaptive immune mechanisms may be potentiated with this combination.

To summarize, it is evident from our studies that AFL changes the immune status of BCC tumors, substantially increasing immune infiltration from the minimal levels that are observed in untreated tumors. The recruitment of innate and adaptive immune cells may play a role in the adjuvant effect of AFL observed on aPD-1 treatment. Whether the immune response observed here is an adaptive anti-tumor response remains to be determined. Further studies could also reveal further details of the innate immune response, including macrophages and natural killer cells. Still, the notion is supported by the increase in the CD8^+^ T-cells and in the MHCII-expressing neutrophils, both of which are backed by previous reports that show a clear role of AFL in boosting the effects of aPD-1 treatment by eliciting an antigen-specific CD8^+^ T-cell mediated immune response [19,24,30].

## 4. Materials and Methods

### 4.1. Animals

Immunocompetent transgenic male and female mice (genotype: Ptch1^+/−^ K14-CreER2 p53^fl/fl^) [43] were used in the study (*n* = 34; ♀: 28, ♂: 6). The mice were kept on a 12 h light/dark cycle in a 23–24 °C facility and were provided with feed and water ad libitum. The study was conducted according to the guidelines of the Declaration of Helsinki and was approved by the Danish Animal Experiments Inspectorate (protocol code 2019-15-0201-01666 of 12 May 2019). Health monitor screening was performed regularly at the facility according to the Federation of Laboratory Animal Science Associations (FELASA) annual tests, and no positive results were found for 45 pathogens (Idexx BioAnalytics, Kornwestheim, Germany).

At the age of 12–20 weeks, the model was induced by dosing 300 µg tamoxifen (Sigma-Aldrich, Munich, Germany) via intraperitoneal injection on three consecutive days, immediately followed by a single dorsal, full-body X-ray irradiation of 4 Gy at 50 kV over a period of 2.05 min (Model D3100, Gulmay Medical, Surrey, Britain). Mice were sedated during irradiation, and their head and tail sections were covered with flexible radiation shielding. Tumors developed within 2–4 months after induction with tamoxifen and X-ray irradiation. Ionizing radiation selectively induced BCC tumors in the PTCH^+/−^ mice [29,50], which we verified histologically in 15 randomly selected tumor samples (Figure 1A).

### 4.2. Study Design

The study was conducted in two parts that assessed (i) tumor response, including mouse survival time, tumor growth rates, and tumor clearance (*n* = 21 mice, 53 tumors), and (ii) immune infiltration (*n* = 13 mice, 19 tumors). The mice were randomized into four intervention groups: aPD-1 monotherapy, AFL monotherapy, aPD-1 with adjuvant AFL, and untreated controls. Mice were included in the tumor response assessment individually, starting treatment, when at least one tumor had reached 3 mm in diameter; mice for immunological analyses were included individually when they had at least one tumor of 5 mm in diameter to provide adequate cells for flow cytometry analysis. Between one and four tumors per mouse were treated at the time of inclusion, depending on the number of tumors with the relevant size that were present. No differences in the number of tumors per mouse (*p* = 0.8450) or in the distribution between sexes (*p* = 0.3490) were observed between interventions.

Treatment was initiated on day 0, and the mice were euthanized on day 60 or when a humane endpoint was reached. For immune analysis, the mice were terminated five days after treatment was initiated. The outcome measures were mouse survival time, tumor growth, tumor clearance, and the recruitment of immune cells (CD45^+^ cells including neutrophils, CD4^+^/CD8^+^ T-cells, and Mo-MDSCs).

### 4.3. Treatment with aPD-1 and AFL

Mice from the aPD-1 and aPD-1+AFL intervention groups received an intraperitoneal injection of 200 µg of anti-PD-1 antibody in saline solution (InVivoMAb, Rat IgG2a kappa, clone 29F.1A12, BioXCell, Lebanon, NH, USA) on days 0, 2, 4, 6, and 8, based on previously published studies [20]. For flow cytometry analysis, the mice received injections on day 0, 2, and 4.

Mice from the AFL and the aPD-1+AFL intervention groups received a single exposure to a 100 mJ/microbeam at 5% density from an Ultrapulse^®^ fractional 10,600 nm CO_2_-laser with a DeepFx handpiece (Lumenis, Inc., Santa Clara, CA, USA) on day 0, with exposure covering the tumor and the immediately surrounding skin tissue (treatment area of 5 × 5 mm–7 × 7 mm depending on tumor size).

### 4.4. Tumor Response

Tumor response was assessed in terms of mouse survival time (controls *n* = 5, aPD-1 *n* = 5, AFL *n* = 6, aPD-1+AFL *n* = 5) as well as growth rates and the tumor clearance time (controls *n* = 11, aPD-1 *n* = 17, AFL *n* = 14, aPD-1+AFL *n* = 11). Tumors were measured 1–2 times per week, with the length and the width of the base of each tumor being recorded in mm. Tumor volumes (mm^3^) were calculated as 0.5 × length × width^2^.

Survival time was defined as the number of days up to 60 from the start of treatment (day 0) to euthanasia, which occurred when the mice reached one of the following humane endpoints: (i) individual tumor size of ≥865 mm^3^ (corresponding to width and length of 12 × 12 mm), (ii) total tumor load (combined size of all tumors on the same mouse) exceeding 2000 mm^3^, or (iii) 20% loss of body weight. In practice, no mice reached humane endpoints (ii) and (iii) during the study.

The growth rates of individual tumors, represented as the growth in mm^3^ per day, were determined by plotting the tumor size (mm^3^) versus the time (days since treatment initiation) followed by curve fitting, with the slope of the curve representing tumor growth rate.

Tumor clearance was defined as no visible tumor or wound at the original tumor site for two independent observations. Tumor clearance is presented for each intervention as the number of cleared tumors relative to the total number of tumors included.

### 4.5. Flow Cytometry

On day five after AFL treatment, the mice were euthanized, after which the tumors were excised (1–4 tumors per mouse). Tumors weighing <100 mg were pooled to obtain a total weight of at least 100 mg per sample, giving a total of 4–7 samples per treatment group time (controls *n* = 7, aPD-1 *n* = 4, AFL *n* = 4, aPD-1+AFL *n* = 4). Tumors were stored at 4 °C in MACS Tissue Storage Solution (Miltenyi Biotec, Bergisch Gladbach, Germany) until further processing later on during the same day. Tumors were minced with scissors and were further digested in murine tumor dissociation enzyme mix (Miltenyi Biotec) and were placed in a shaking water bath at 37 °C for 40 min. To obtain a single-cell suspension, the enzyme-treated tumor tissues were mechanically dispersed by filtering twice through a 70 µm cell strainer. The total number of cells in each sample was determined using the MUSE Cell Analyzer (Merck Millipore, Burlington, MA, USA) according to the manufacturer’s instructions. Samples with a yield above 3 × 10^6^ cells were included in further analyses. Cells were resuspended in 50 µg/mL purified rat anti-mouse CD16/CD32 (Becton Dickinson, Franklin Lakes, NJ, USA) and were incubated for 5 min. on ice to block the Fc-receptors before the samples were surface stained specific antibodies (Table S1) and eBioscience™ Fixable Viability Dye eFluor™ 780 (for 30 min at 4 °C). The samples were filtered through a 70 µm filter before acquisition on a BD LSRFortessa X-20 flow cytometer (BD Biosciences). Single-color stained UltraComp eBeads Plus Compensation beads (Invitrogen, Waltham, MA, USA) and single-stained tumor samples were included to compensate for spectral spillover. Flow cytometry data were analyzed using FlowJo v10.7 (FlowJo LLC, Ashland, OR, USA). Immune cells were defined as CD45^+^ cells. In addition, the neutrophils were defined as CD11b^+^ CD11c^−^ Ly-6C^int^ Ly-6G^+^; Mo-MDSCs were defined as CD11b^+^ CD11c^−^ Ly-6G^−^ or Ly-6C^high^; and T-cells were defined as CD11b^−^ CD11c^−^ and either CD4^+^ or CD8^+^ cells. Further details on the gating strategy are provided in Figure S3.

### 4.6. Statistics

Mouse survival time was analyzed using a Kaplan–Meier plot, and the groups were compared using Mantel-Cox log-rank tests. Tumor growth rates, clearance, and immune infiltration were compared using the Mann–Whitney test. Differences were considered significant when *p*-values were less than 0.05. Data are presented as medians and interquartile ranges. Statistical analyses were performed using GraphPad Prism 9.0.0 (GraphPad Software, San Diego, CA, USA) and IBM SPSS 25.0 (SPSS Inc., Chicago, IL, USA).

## 5. Conclusions

In conclusion, in this explorative study, we found that AFL can serve as an adjuvant for the aPD-1 treatment of BCCs and that it can substantially improve tumor immune infiltration and outcome in a clinically relevant BCC mouse model. Altogether, this highlights the therapeutic potential of a locally applied adjuvant AFL for enhancing the efficacy of systemic immunotherapy, although the results will need to be confirmed in a clinical trial.

## Data Availability

The data that support the findings of this study are available from the corresponding author upon reasonable request.

## Supplementary Materials

The following are available online at https://www.mdpi.com/article/10.3390/cancers13246326/s1, Figure S1: CD4^+^:CD8^+^ T-cell ratios compared between interventions. Figure S2: Treatments do not change MHCII expression level in monocytic myeloid-derived suppressor cells. Figure S3: Flow cytometry gating strategy. Table S1: Antibody list.
