# Supplementary material for: Anti-PD-1 Therapy with Adjuvant Ablative Fractional Laser Improves Anti-Tumor Response in Basal Cell Carcinomas
Source: Cancers (Basel). 2021 Dec 16;13(24):6326. doi: 10.3390/cancers13246326 (PMC8699063; doi:10.3390/cancers13246326)
Supplement: Supplementary file 1 [file cancers-13-06326-s001.zip › cancers-1490952-supple.pdf]

Supplementary figures and tables

Antigen	Clone	Fluorophore	Supplier
CD45	30-F11	BUV395	BD Biosciences
CD8	53-6.7	BUV 737	BD Biosciences
I-A/I-E	M5/114.15.2	BV421	BD Biosciences
CD11b	M1/70	BV480	BD Biosciences
CX3CR1	BSA011F11	BV650	Biolegend
CD25	PC61	BV711	BD Biosciences
CD11c	HL3	BV786	BD Biosciences
CD3	17A2	FITC	BD Biosciences
CD64	X54-5/7.1	PE	Biolegend
CD4	GK1.5	PE-Cy7	BD Biosciences
Ly-6C	AL-21	APC	BD Biosciences
Ly-6G	1A8	Alexa Fluor 700	Biolegend

Supplementary Table S1 — Antibody list

List of antibodies used for flow cytometry analysis in our study.

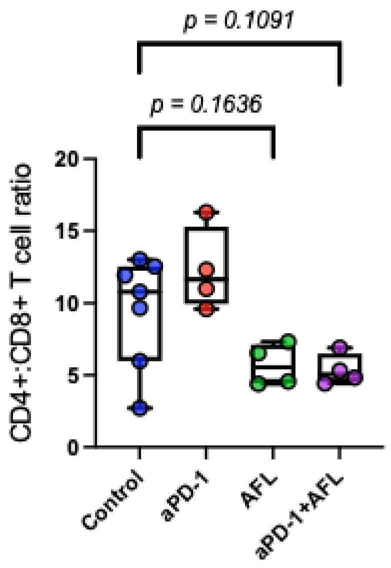

Supplementary Figure S1. CD4$^+$:CD8$^+$ T-cell ratios compared between interventions

Number of CD4$^+$ T-cells relative to CD8$^+$ T-cell counts (in percentage). aPD-1: Programmed cell death-1 immune checkpoint inhibitor. AFL: ablative fractional laser. Group size n = 4–7.

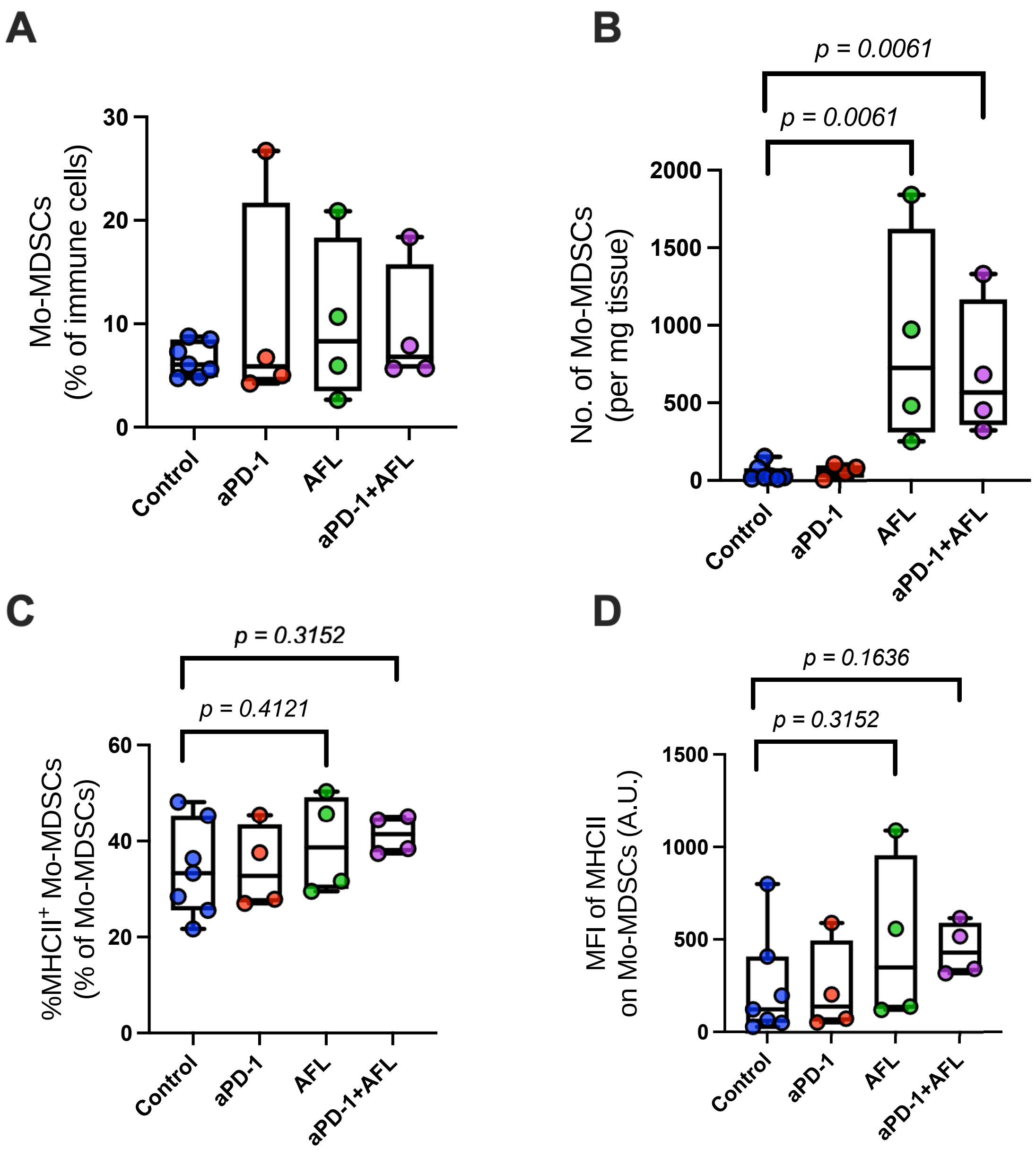

Supplementary Figure S2. Treatments do not change MHCII expression level on monocytic myeloid-derived suppressor cells

(A) Relative number of Mo-MDSCs to total immune cells (in percentage). **(B)** Absolute number of Mo-MDSCs per mg of tumor tissue. **(C)** Proportion of Mo-MDSC positive for major histocompatibility complex class II (MHCII) and **(D)** the median fluorescent intensity (MFI) of MHCII on Mo-MDSCs. Mo-MDSCs were defined as $CD11b^{+}$ $CD11c^{-}$ Ly-$6G^{-}$, Ly-$6C^{high}$. aPD-1: Programmed cell death-1 immune checkpoint inhibitor. AFL: ablative fractional laser. Group size n = 4–7.

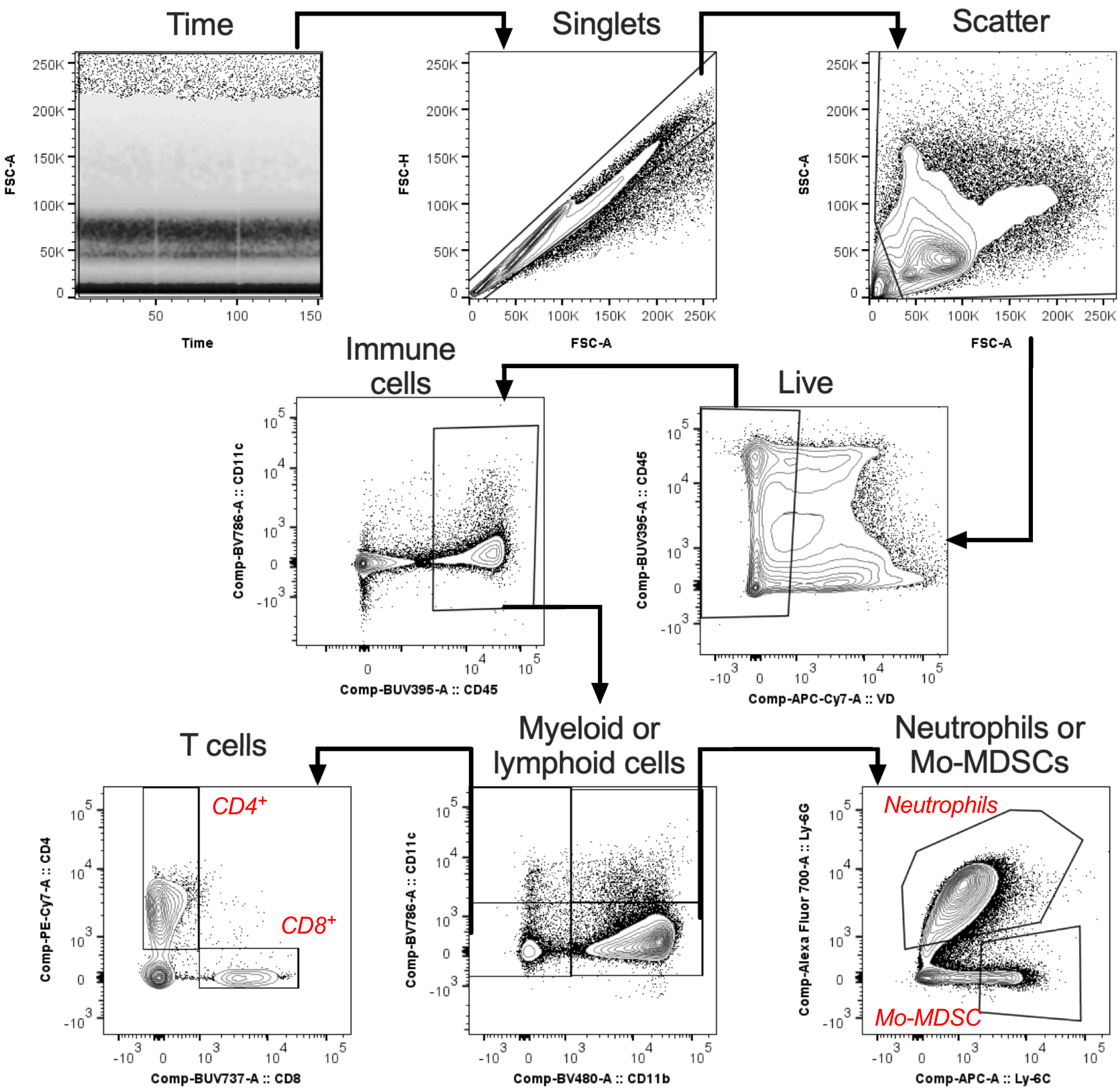

Supplementary Figure S3. Flow cytometry gating strategy

Contour plots representative of gating strategy for acquired flow cytometry data. All immune cells were gated on *Time, singlets, scatter, viability and CD45*$^{+}$. Mo-MDSC: monocytic myeloid-derived suppressor cells
